# Use of the Common Marmoset to Study *Burkholderia mallei* Infection

**DOI:** 10.1371/journal.pone.0124181

**Published:** 2015-04-10

**Authors:** Tomislav Jelesijevic, Shawn M. Zimmerman, Stephen B. Harvey, Daniel G. Mead, Teresa L. Shaffer, D. Mark Estes, Frank Michel, Frederick D. Quinn, Robert J. Hogan, Eric R. Lafontaine

**Affiliations:** 1 Department of Infectious Diseases, College of Veterinary Medicine, University of Georgia, Athens, Georgia, United States of America; 2 Department of Population Health, College of Veterinary Medicine, University of Georgia, Athens, Georgia, United States of America; 3 Department of Microbiology, Franklin College of Arts and Sciences, University of Georgia, Athens, Georgia, United States of America; 4 Department of Veterinary Biosciences and Diagnostic Imaging, College of Veterinary Medicine, University of Georgia, Athens, Georgia, United States of America; University of Toledo School of Medicine, UNITED STATES

## Abstract

*Burkholderia mallei* is a host-adapted bacterium that does not persist outside of its equine reservoir. The organism causes the zoonosis glanders, which is endemic in Asia, Africa, the Middle East and South America. Infection by *B*. *mallei* typically occurs via the respiratory or percutaneous route, and the most common manifestations are life-threatening pneumonia and bacteremia. Glanders is difficult to diagnose and requires prolonged antibiotic therapy with low success rates. There is no vaccine to protect against *B*. *mallei *and there is concern regarding its use as a biothreat agent. Thus, experiments were performed to establish a non-human primate model of intranasal infection to study the organism and develop countermeasures. Groups of marmosets (*Callithrix jacchus*) were inoculated intranasally with *B*. *mallei* strain ATCC 23344 and monitored for clinical signs of illness for up to 13 days. We discovered that 83% of marmosets inoculated with doses of 2.5 X 10^4^ to 2.5 X 10^5^ bacteria developed acute lethal infection within 3–4 days. Signs of disease were severe and included lethargy, inappetence, conjunctivitis, mucopurulent and hemorrhagic nasal discharges, and increased respiratory effort with abdominal lifts. *Burkholderia mallei* was cultured from the lungs, spleen and liver of these animals, and pathologic examination of tissues revealed lesions characteristic of glanders. Challenge experiments also revealed that 91% of animals infected with doses ranging from 25 to 2.5 X 10^3^ bacteria exhibited mild non-specific signs of illness and were culture negative. One marmoset inoculated with 2.5 X 10^3^ organisms developed moderate signs of disease and reached humane end-points 8 days post-infection. The liver and spleen of this animal were colonized with the agent and pathological analysis of tissues showed nasal, splenic and hepatic lesions. Taken together, these data indicate that the marmoset is a suitable model to study respiratory infection by *B*. *mallei*.

## Introduction

*Burkholderia mallei* is a non-motile Gram negative bacillus that does not persist in the environment outside of its natural equine reservoir. The organism causes the highly contagious and debilitating zoonotic disease glanders, which primarily affects horses, mules and donkeys. Glanders is an old disease, having first been described by Aristotle in 330 B.C. Other less commonly known names that have been used throughout history to describe the disease include equinia, malleus, droes and farcy. Though now considered exotic in North America and Europe, glanders once was endemic in these regions. Early settlers introduced the disease in the U.S. via the import of infected horses. By the 2^nd^ half of the 19^th^ century, glanders was widespread due to the Civil War, when it was readily transmitted to thousands of cavalry horses passing through remount stations. In response to this epidemic, industrial countries implemented vigorous eradication programs that entailed financial incentive to owners, destruction of infected animals, and disinfection of affected facilities. Great Britain was first to eradicate the disease in 1928, followed by the U.S. in 1934, Canada in 1938, and most European countries by 1965 [1–9]. Despite the success of eradication efforts, naturally occurring glanders is still found in parts of Asia, Africa, the Middle East and South America. The disease is closely monitored by the World Organization for Animal Health (OIE) as it is considered a serious safety and biosecurity threat.

Humans, goats, dogs, cats, rabbits, and carnivorous predators living in the vicinity of equids with glanders, have been naturally infected. Camels have also been reported to harbor the disease and have been linked to zoonotic transmission [1–7, 10–13]. In humans, infection generally occurs via the respiratory route or direct invasion of punctured skin after contact with infected animals. Disease progression and pathology in humans and horses are similar, though the presentation of any 2 cases in the same species, even if related by direct transmission, may vary. Clinical manifestations include fever, myalgia, fatigue, lymphadenopathy, pneumonia and necrosis of the tracheobronchial tree. Pustular skin lesions and development of multiple abscess-forming nodules, known as farcy, are also common. Most patients become bacteremic, and *B*. *mallei* bacilli disseminate to the liver, spleen and lymph nodes where it rapidly causes necrotizing abscesses [1–7, 10, 14–18]. The disease is difficult to identify and a conclusive diagnosis requires culturing *B*. *mallei* from patients. The mortality rate is high (up to 50%) despite aggressive antimicrobial therapy. *Burkholderia mallei* is resistant to many antibiotics, which limits treatment options. The recommended two-phase treatment process involves the use of ceftazidime and meropenem (intensive phase), followed by TMP-SMX and co-amoxiclav (eradication phase) for several weeks [19]. Response to treatment is slow, and unless treated immediately, eradication of the agent can be difficult as patients are prone to relapses characterized by prolonged bouts of remission and recrudescence. There is currently no vaccine available to reduce the risk of infection by *B*. *mallei*.

Though human cases of glanders are rare, there is legitimate concern that *B*. *mallei* might be used as a bioweapon because it has been utilized in this manner on multiple occasions [1, 5, 20–25]. During World War I, Germany implemented a bold biological sabotage campaign in several countries including the U.S., Russia, Romania, France and Mesopotamia. For example, horses, mules, and other livestock being shipped from the U.S. to allies were inoculated with cultures of *B*. *mallei*. German agents also infected thousands of mules in Mesopotamia with the organism, and a German agent was arrested in Russia with similar intentions in 1916. Between 1932 and 1945, the Japanese deliberately infected horses, civilians, and prisoners of war with glanders at the Pin Fan Institute in occupied Manchuria. More recently (1980s), the Soviet Union purportedly weaponized *B*. *mallei* and used it against opposition forces in Afghanistan [24]. For these reasons, the U.S. Federal Select Agent Program classifies *B*. *mallei* as a Tier 1 agent, and the development of medical countermeasures (MCM) against the organism is highly desirable.

Several animal models have been developed to study glanders including hamsters, mice and horses [26–31]. These models have been crucial to our current understanding of pathogenesis by *B*. *mallei* and provide excellent platforms for developing MCM (*i*.*e*. identification and characterization of targets). In contrast, there have been very few reports describing the use of non-human primates as surrogates to study glanders and validate lead MCM candidates [32–34]. To address this, we performed experiments to establish a model of intranasal infection for *B*. *mallei* using the common marmoset. Our data indicate that this species is a suitable surrogate to study glanders.

## Materials and Methods

### Bacterial strain and growth conditions

The *B*. *mallei* strain ATCC 23344 [35] was used in this study. To prepare the inoculum for infection, the organism was cultured on Brucella broth agar (BD-BBL) supplemented with 5% (vol/vol) glycerol for 42 hr at 37°C. These plate-grown bacteria were suspended in Phosphate-Buffered Saline (PBS) to an optical density of 250 Klett units using a Klett Colorimeter (Scienceware). Following this, the suspension was serially diluted and 100 μL aliquots were immediately spread onto agar plates to determine the number of colony forming units (CFU) in the inoculum. Fifty μL of serial dilutions 10^-2^ (250,000 CFU), 10^-3^ (25,000 CFU), 10^-4^ (2,500 CFU), 10^-5^ (250 CFU), and 10^-6^ (25 CFU) were used to infect animals.

### Experimental animals and intranasal infection procedures

Both male and female, healthy, sexually mature common marmosets (*Callithrix jacchus*) were obtained from Worldwide Primates Inc. The animals were >36 months old and weighed 220–430 grams at the time of infection, and were acclimatized to their new surroundings for at least two weeks. Prior to inoculation, the animals were anesthetized in their nest boxes with inhalant isoflurane gas until a respiratory rate of 10–12 breaths per minute was achieved. Once anesthetized, the animals were held in supine position and droplets of *B*. *mallei* suspension (5–10 μL) were delivered to the right nostril using a sterile filtered tip attached to a P200 Pipetman. The droplets were inhaled through the nasal passageways, and a total volume of 50 μL of bacterial suspension was administered.

Marmosets were housed individually in stainless steel cages (16.625” W x 18.75” D x 30.625” H, open wirebar-front and top) positioned to allow visual and vocal communication. The animals were provided various forms of environmental enrichment including wooden perches, mirrors, teethable circular link baby toys, and Kong puzzle toys filled with forage items (dried fruits or vegetables, seeds, nuts, and miniature marshmallows). Water and food were provided *ad libitum*. The animals were fed a balanced commercial primate diet (ZuPreem) supplemented with various food items (fruit, nuts, yogurt, eggs, rice, pasta) in order to recreate their previous diet at the Worldwide Primates Inc. Therapeutic intervention with antibiotics and/or analgesics was not provided as such treatments would have affected the experimental outcomes of the study. Infected animals were monitored every 8 hr.

Humane end-points were strictly observed. Marmosets exhibiting signs of moderate to severe discomfort were euthanized. The following scoring system was used to determine if euthanasia was appropriate: 3 points: complete anorexia and/or lack of water consumption for 24 hr; 3 points: non-responsiveness to external stimuli, recumbency, marked difficulty breathing (dyspnea); 2 points: a "dull" appearance, manifested in part by a reduced responsiveness to external stimuli or lethargic movements in the cage; 2 points: failure to enter the nestbox or climb onto a resting perch; 2 points: failure to consume water for 24 hr; 1 point: mild changes in respiration, such as slightly increased difficulty or an increased respiratory rate; 1 point: inappetence for 1 day. Any animal with a point score ≥ 3 was humanely euthanized. This was accomplished by anesthetizing marmosets in their nest boxes using inhalant isoflurane gas. Once anesthetized, the animals were administered a dose of 10–20 mg/kg ketamine intramuscularly and then euthanized by intracardiac injection of a pentobarbital dose of 240 mg/kg. This procedure is in accordance with the AVMA Guidelines for the Euthanasia of Animals. The median lethal dose (LD_50_) value was calculated according to Reed and Muench [36], and is defined as the dose where 50% of the animals reached humane end-points.

### Post-mortem analysis

A board-certified veterinary anatomic pathologist performed post-mortem examination of tissues and organs from all animals.

### Bacteriology

Samples of lung, liver, spleen, and trachea were aseptically collected for bacterial culture prior to processing the tissues for cytology and histology. These samples were weighed, homogenized with 15-mL disposable tissue grinders (Fisherbrand), serially diluted, and plated on agar medium to calculate the number of viable *B*. *mallei* bacteria in tissues. To suppress growth of the marmoset normal flora, agar plates containing 8 μg/mL Polymixin B (MP Biomedicals), 2 μg/mL Bacitracin (MP Biomedicals) and 5 μg/mL Cyclohexamide (MP Biomedicals) were utilized.

### Cytology

Tissue imprints of lung, liver, and spleen were prepared on glass slides, air-dried for 10 min, fixed with 100% anhydrous methanol, and stained with modified Wright-Giemsa using an automated Aerospray Hematology Slide Stainer (Wescor Biomedical Systems). Cytologic evaluation was performed by a board-certified veterinary clinical pathologist using an Olympus BX51 light microscope (Olympus Corporation). Images were captured using an Olympus DP71 camera and software (Olympus Corporation).

### Histology

Tissues selected for histology included sagittal sections of the right nasopharynx, sections of all lobes of the lungs, trachea, submandibular and tracheobronchial lymph nodes, liver, spleen, kidneys, adrenal glands, and representative sections of the gastrointestinal tract (stomach, duodenum, jejunum, ileum, cecum, colon). The tissues were submersed in 10% buffered formalin (Fisher Chemical) for 3 weeks, cut, placed into histology cassettes for biopsy samples (Leica BIOSYSTEMS), and fixed in 10% buffered formalin for an additional 2 days. For nasopharyngeal tissues, the animals' heads were bisected and placed into decalcification solution (Leica BIOSYSTEMS) for 5 hr prior to cutting, placing into cassettes, and submersing into 10% buffered formalin. After verifying that fixed tissues no longer contained live *B*. *mallei*, the samples were taken out of the BSL3 laboratory, embedded in paraffin, cut into 3 μm-thick sections, and stained with hematoxylin and eosin (H&E). Histologic evaluations were performed by a board-certified veterinary anatomic pathologist using an Olympus BX41 microscope (Olympus Corporation). Images were captured using an Olympus DP71 camera and software (Olympus Corporation).

Selected tissues were stained using a modified Gram stain method developed by the Histology Laboratory at The University of Georgia College of Veterinary Medicine, which identifies bacteria as Gram positive (purple-black) or Gram negative (dark pink-red). Selected tissues were also concurrently processed for examination by immunofluorescence microscopy. Briefly, tissue sections were incubated in a 10 mM sodium citrate buffer (pH 6.2) for 10 min at a temperature of 120°C to unmask antigens. Following this high-temperature antigen retrieval step, the tissues were washed, permeabilized with a solution containing 0.05% Triton X-100 for 20 min at room temperature, and probed for 1 hr with convalescent serum from mice that survived acute *B*. *mallei* infection. The serum, which was obtained in the context of a previous study [31], was used at a dilution of 1:100. Next, the tissues were washed to remove excess primary antibodies and incubated at room temperature for 1 hr with a goat anti-mouse IgG (H+L) antibody labeled with AlexaFluor 488 (Life Technologies) at a dilution of 1:100. After washing off unbound secondary antibody, the tissues sections were treated with Prolong Gold Antifade Mountant reagent containing DAPI (Life Technologies) and examined by microscopy with a Nikon Eclipse Ti confocal microscope. Images were captured and analyzed with the Nikon NIS-Elements software. Phosphate-buffered saline (pH 7.4) supplemented with 0.05% Tween 20 and 1% normal goat serum (SIGMA-ALDRICH) was used to wash tissues and dilute antibodies.

### Compliance and animal research ethic statement

Preparation of the inoculum for infection and processing of tissues containing live *B*. *mallei* bacilli were performed inside a Class II Biosafety Cabinet (BSC) in a BSL3 laboratory. Animals infected with *B*. *mallei* were housed in wire front and top cages within an ABSL3-Ag room, which served as primary containment (air HEPA-filtered in and out, 15 complete air changes per hour), at The University of Georgia Animal Health Research Center. The animal facility is part of the University of Georgia’s program that is accredited by the Association for Assessment and Accreditation of Laboratory Animal Care, International (AAALAC-I). Post-mortem examination and tissue collection were performed in a BSL3 necropsy suite equipped with a Class II BSC. All experiments were performed in compliance with the rules and regulations of the U.S. Federal Select Agent Program and were approved by The University of Georgia Institutional Biosafety Committee (IBC) and Institutional Animal Care and Use Committee (IACUC). Animal experiments were carried out in strict accordance with the recommendations in the Guide for the Care and Use of Laboratory Animals of the National Institutes of Health. All efforts were made to minimize animal suffering. Anesthesia and euthanasia procedures were performed by trained personnel including board-certified veterinarians, animal care staff, and research team members. The IACUC approval number covering this specific study is AUP A2013 01-018-Y2-A2.

## Results

### Median lethal dose determination and clinical findings

*Burkholderia mallei* infection generally occurs via the respiratory or percutaneous route. The respiratory route of infection, and subsequent pulmonary disease, are of particular concern with respect to the use of *B*. *mallei* as a biothreat agent because the airways are the most likely route of entry for the bacterium during a biologic attack. For these reasons, we developed a marmoset model of intranasal infection to study the organism. Five groups of animals were inoculated with increasing doses of strain ATCC 23344 and monitored for clinical signs of illness for a period of up to 13 days. Two uninfected marmosets were included as controls and were housed in the same room with the infected animals. The results are summarized in Table 1. Based on these data, the calculated median lethal dose for the organism is 9,953 CFU. This value is higher than that reported for other models of respiratory infection. Using a similar intranasal route of inoculation, the LD_50_ in mice has been reported to be 820 CFU [37]. Nebulization of the organism into the murine lungs with a MicroSprayer device produced roughly the same value [31]. The use of a mouse whole body aerosol model yielded LD_50_ doses of 1,000–1,800 CFU [29, 38, 39].

**Table 1:** Marmoset groups, identifiers, inoculating doses, clinical presentations, and bacterial loads in selected tissues.

Identifier	Inoculating dose (CFU)	End point^**¥**^	Clinical presentation	Survival	Lungs^**¶**^	Spleen^**¶**^	Liver^**¶**^	Trachea^**¶**^
171	25	13	Mild, non-specific		-	-	-	-
172	25	13	Mild, non-specific	4/4	-	-	-	-
174	25	13	Mild, non-specific		-	-	-	-
186	25	13	Mild, non-specific		-	-	-	-
189	250	12	Mild, non-specific		-	-	-	-
191	250	12	Mild, non-specific	4/4	-	-	-	-
194	250	12	Mild, non-specific		-	-	-	-
196	250	12	Mild, non-specific		-	-	-	-
225	2,500	11	Mild, non-specific		-	-	-	-
968	2,500	11	Mild, non-specific	3/4	-	-	-	-
D65	2,500	11	Mild, non-specific		-	-	-	-
D37	2,500	8*	Moderate, specific		-	2.7x10^3^	1.3x10^3^	-
109	25,000	4*	Moderate, specific		-	1.8x10^4^	5.6x10^3^	-
755	25,000	4*	Moderate, specific	1/3	1.6x10^4^	1.9x10^4^	2.3x10^3^	-
135	25,000	12	Mild, non-specific		-	-	-	-
F48	250,000	4*	Severe, specific		3.6x10^4^	7.2x10^4^	6.1x10^3^	-
55B	250,000	3*	Severe, specific	0/3	ND	ND	ND	ND
544	250,000	3**	Severe, specific		ND	ND	ND	ND

-: *B*. *mallei* not recovered from tissues

ND: not determined

^¥^: days post-infection

*: animal reached humane end point and was euthanized

**: animal was unexpectedly found dead

^¶^: Bacterial burden (CFU/gr of tissues)

Given that the common marmoset had not been previously used to study respiratory infection by *B*. *mallei*, considerable effort was put into documenting the clinical presentation of disease. As expected, the severity and onset of clinical signs correlated with the bacterial dose administered. Marmosets in the low dose groups (25 and 250 CFU) displayed mild and non-specific signs of illness. At day 7 post-infection, the animals stopped grooming and by day 10, most exhibited withdrawn behavior (hiding, moderately reduced vocalization, limp tails), had decreased appetite and reduced activity, and developed dry and crusted nostrils. One marmoset from each group also displayed mild unilateral conjunctivitis at the end point of the study (172 and 194, Table 1). Animals in the medium dose group (2,500 CFU) developed a similar disease presentation. By day 10, nearly all exhibited crusted nostrils and were often observed wiping their faces against their perches or with their hands, indicating the presence of nasal discharge. One marmoset, 225, was confirmed to have a clear nasal discharge when euthanized at the end point of the study. Another marmoset was observed sneezing on multiple occasions and quickly developed unilateral conjunctivitis (D37, Table 1). On day 8 post-infection, this animal presented with an increased respiratory rate and was euthanized, having reached humane experimental end points.

Two marmosets in the medium-high dose group (25,000 CFU) displayed moderate symptoms of disease. By day 4, both reached humane experimental end points as they exhibited rough coats, unilateral conjunctivitis, clear nasal discharges, inappetence, lethargy, and increased respiratory rates (109 and 755, Table 1). The third animal in this group (135, Table 1) only showed mild and non-specific signs of illness (reduced grooming, moderately decreased vocalization, limp tail).

Marmosets in the high dose group (250,000 CFU) rapidly developed severe signs indicative of glanders. Two days post-infection, all three animals stopped grooming, were inappetent, and their water intake was reduced. One marmoset unexpectedly died ~60 hr post-challenge (544, Table 1). This animal had an increased respiratory rate and showed evidence of ocular/nasal discharge (wet face), but was mobile and responsive to stimulation 52 hr post-infection, and thus had not met the humane endpoint criteria. Another marmoset had clear ocular and nasal discharges as well as unilateral conjunctivitis 72 hr post-challenge (55B, Table 1). Eight hours later, this animal was euthanized, as it became moribund and exhibited hemorrhagic nasal discharge, increased respiratory effort with abdominal lifts, and a head tilt. The third marmoset in this group was euthanized on day 4 after developing overnight a mucopurulent discharge from the right nostril, unilateral conjunctivitis, lethargy, inappetence, and increased respiratory rate (F48, Table 1).

The two uninfected control animals exhibited mild and non-specific signs of illness attributable, presumably, to the BSL3 environment. These included brief periods of decreased appetite and mildly withdrawn behavior (limp tail, reduced vocalization), occasional soft stools, and rough unkempt coat at the end point of the study.

### Bacteriology findings

To examine replication and dissemination of *B*. *mallei*, we collected tissues from infected animals at the indicated end points and determined bacterial loads in the lungs, spleen, liver, and trachea. The results are shown in the last 4 columns of Table 1. All trachea samples and tissues collected from the two uninfected control marmosets (data not shown) were culture negative. The organism was not recovered from the tissues of animals in the low dose groups (25 and 250 CFU), which is consistent with their non-specific clinical presentation. Likewise, tissues from marmosets 225, 968 and D65 in the medium dose group (2,500 CFU) were culture negative. The fourth marmoset in this group, D37, was colonized with *B*. *mallei* in the spleen and liver, which correlates with the overt signs of illness displayed by the animal on day 8 post-infection.

The two marmosets in the medium-high dose group (25,000 CFU) exhibiting moderate symptoms of infection were colonized with *B*. *mallei* (109 and 755, Table 1). The spleen and liver of both animals were culture positive, but only one marmoset was colonized in the lungs. *Burkholderia mallei* bacilli were not recovered from tissues of the third animal in this group, 135, which is consistent with the mild and non-specific signs of illness displayed by the marmoset throughout the study. Tissues from only one animal in the high dose group (250,000 CFU) were processed for culture, and the results show colonization of the spleen, liver and lungs (F48, Table 1).

### Cytology findings

**Fig 1:**
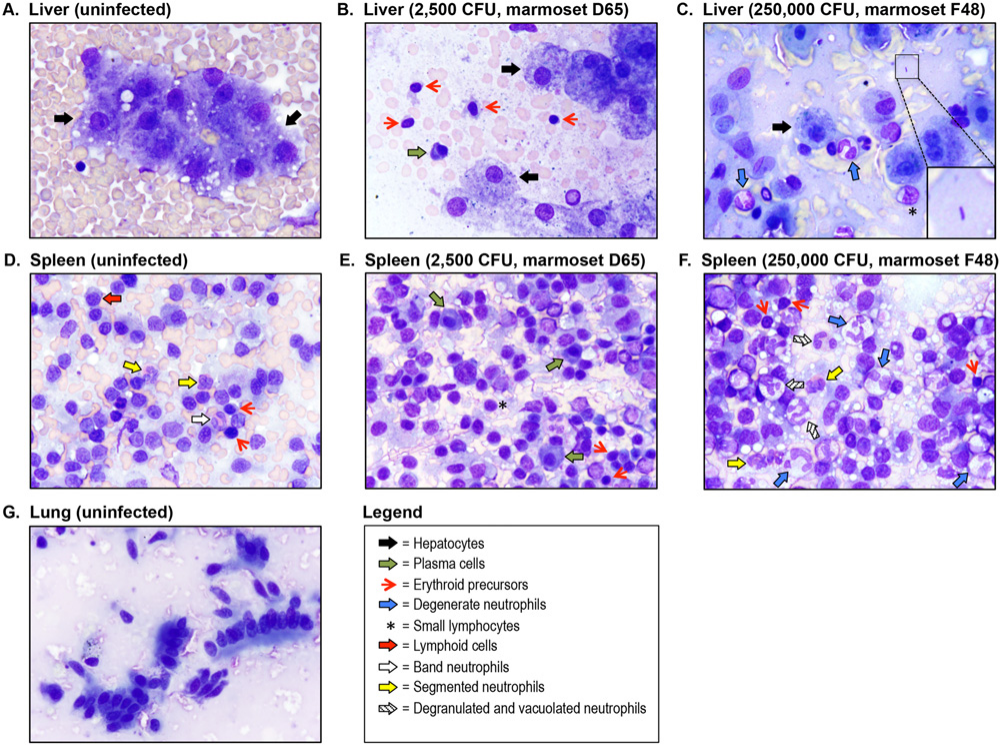
Cytologic examination of liver, lung and spleen impression smears. Tissue imprints from control and infected animals were made on glass slides, fixed with methanol, air-dried, stained with modified Wright-Giemsa, examined by light microscopy and photographed. Representative fields are shown. Panel A shows a liver sample from an uninfected control marmoset. The sample was high in cellularity and consisted of clumps of well-differentiated hepatocytes (black block arrows). Panel B shows a liver sample from marmoset D65, which was infected with a medium dose of *B*. *mallei* (2,500 CFU). The sample was high in cellularity and consisted of well-differentiated hepatocytes (black block arrows), admixed with increased numbers of small lymphocytes, plasma cells (green block arrow), and erythroid precursors (red arrows). Panel C shows a liver sample from marmoset F48, which was infected with a high dose of *B*. *mallei* (250,000 CFU). It consisted primarily of large numbers of degenerate neutrophils (blue block arrows) admixed with well-differentiated hepatocytes (black block arrows), some small lymphocytes (asterisk), extracellular bacilli (inset), and disrupted cellular debris. Panel D shows a splenic sample from an uninfected control marmoset. The sample was high in cellularity and consisted primarily of lymphoid cells (red block arrow) and erythroid precursors (red arrows) admixed with occasional heavily granulated band neutrophils (white block arrow) and segmented neutrophils (yellow block arrows). Panel E shows a splenic sample from marmoset D65, which was infected with a medium dose of *B*. *mallei* (2,500 CFU). The sample was high in cellularity and contained plasma cells (green block arrows), small lymphocytes (asterisk), and erythroid precursors (red arrows). Panel F shows a splenic sample from marmoset F48, which was infected with a high dose of *B*. *mallei* (250,000 CFU). The sample was high in cellularity and consisted of many degenerate neutrophils (blue block arrows), degranulated and vacuolated neutrophils (shaded block arrows), segmented neutrophils (yellow block arrow) and an increased number of erythroid precursors (red arrows). Panel G shows a lung sample from an uninfected control marmoset. The sample was high in cellularity and consisted of both individualized and clumped, well-differentiated, ciliated, columnar epithelial cells.

To investigate pathologic changes induced by *B*. *mallei* during infection, tissue imprints (liver, spleen, lungs) were prepared at the indicated end points and examined by microscopy. The cytology findings are summarized in Table 2 and images of representative impression smears are shown in Fig 1. Consistent with clinical and bacteriology findings, the tissues of animals in the low dose groups (25 and 250 CFU) did not show cytologic evidence of infection and looked identical to the samples from uninfected controls. The liver impression smears were high in cellularity and contained clumps of well-differentiated hepatocytes (Fig 1A). The splenic imprints also were high in cellularity and consisted of many lymphoid cells, minimal extramedullary hematopoiesis (*i*.*e*. presence of erythroid, myeloid, and megakaryocytic precursor cells), and occasional segmented neutrophils with normal morphology (Fig 1D). The lung specimens were highly cellular and contained well-differentiated ciliated columnar epithelial cells (Fig 1G).

**Table 2:** Marmoset identifiers, groups, and cytology findings.

Identifier	Inoculating dose (CFU)	Lungs	Spleen	Liver
171	25	Normal	Normal	Normal
172	25	Normal	Normal	Normal
174	25	Normal	Normal	Normal
186	25	Normal	Normal	Normal
189	250	Normal	Normal	Normal
191	250	Normal	Normal	Normal
194	250	Normal	Normal	Normal
196	250	Normal	Normal	Normal
225	2,500	Normal	Normal	Normal
968	2,500	Normal	Normal	Normal
D65	2,500	Normal	Moderate LH	Mild-moderate LH
D37	2,500	Normal	Mild-moderate suppurative inflammation with degenerate neutrophils	Mild-moderate suppurative inflammation with degenerate neutrophils and extracellular bacilli
109	25,000	Normal	Moderate suppurative inflammation, moderate LH and EMH	Normal
755	25,000	Normal	Moderate suppurative inflammation, moderate LH and EMH	Normal
135	25,000	Normal	Moderate-marked LH and EMH	Mild-moderate EMH
F48	250,000	Normal	Severe suppurative inflammation, severe LH and EMH	Severe suppurative inflammation, severe LH and EMH, degenerate neutrophils with extracellular bacilli
55B	250,000	ND	ND	ND
544	250,000	ND	ND	ND

EMH = extramedullary hematopoiesis, LH = lymphoid hyperplasia, ND = not determined

The samples obtained from marmosets 225 and 968 in the medium dose group (2,500 CFU) appeared normal, correlating with their unremarkable clinical presentation and culture negative status (Table 1). Interestingly, the liver and splenic impression smears from marmoset D65, which was also culture negative and exhibited non-specific signs of illness, showed lymphoid hyperplasia (Fig 1B and 1E). The smears contained increased numbers of medium-sized lymphocytes, plasma cells, and even rare Mott cells, suggesting antigen stimulation. Samples from the fourth marmoset in the medium dose group, D37, were clearly indicative of infection (Table 2). The liver imprint showed extracellular bacilli and suppurative inflammation characterized by many degenerate neutrophils intimately associated with hepatocytes. Degenerate neutrophils are essentially dying segmented neutrophils that possess swollen and pale nuclei. These cells typically form in toxic microenvironments due to the presence of bacterial endotoxin (*i*.*e*. LPS), which perforates the nuclear and cellular membranes of the neutrophil and ultimately inhibits the cell’s ability to control water homeostasis. The splenic sample from marmoset D37 also contained numerous degenerate neutrophils. These findings are consistent with the moderate symptoms of glanders displayed by the animal on day 8 post-challenge and colonization of its spleen and liver with *B*. *mallei* (Table 1). The lung impression smears collected from all 4 marmosets in the medium dose group were within normal limits.

The spleen samples from marmosets 109 and 755 in the medium-high dose group (25,000 CFU) exhibited suppurative inflammation. Neutrophil morphology in these samples ranged from normal to vacuolated and degranulated. Neutrophil vacuolation and degranulation typically occur during inflammation and signify the release of bactericidal molecules (degradative enzymes, antimicrobial peptides, reactive oxygen species). The samples also showed moderate lymphoid hyperplasia as well as moderate extramedullary hematopoiesis (Table 2). These findings are consistent with the moderate symptoms of disease displayed by the animals on day 4 post-challenge and colonization of their spleens with *B*. *mallei* (Table 1). The liver impression smears from marmosets 109 and 755 did not show cytologic evidence of infection, which was unexpected given that *B*. *mallei* was cultured from these tissues. The third animal in the medium-high dose group, 135, did not show signs of illness and was culture negative (Table 1). Conversely, spleen and liver imprints from this marmoset demonstrated marked lymphoid hyperplasia and moderate extramedullary hematopoiesis, which indicate antigen stimulation and increased hematopoietic demand, respectively (Table 2). The lung impression smears from all 3 animals in the medium-high dose group appeared normal.

Tissue impressions from only one marmoset in the high dose group (250,000 CFU) were examined and were clearly indicative of bacterial infection (F48, Table 2). Representative images of the liver and spleen imprints are shown in Fig 1C and 1F, respectively, and demonstrate severe suppurative inflammation characterized by a large number of degenerate neutrophils intimately associated with parenchymal cells. Many extracellular, medium-sized bacilli were observed in the background of the liver sample (see inset in Fig 1C). Both tissues also showed marked extramedullary hematopoiesis and severe lymphoid hyperplasia. These findings are consistent with the severe clinical signs of glanders displayed by this animal on day 4 post-challenge, and colonization of its spleen and liver with *B*. *mallei* (Table 1). Consistent with the other experimental groups, the lung impression from marmoset F48 was cytologically unremarkable.

### Gross and histology findings

**Fig 2:**
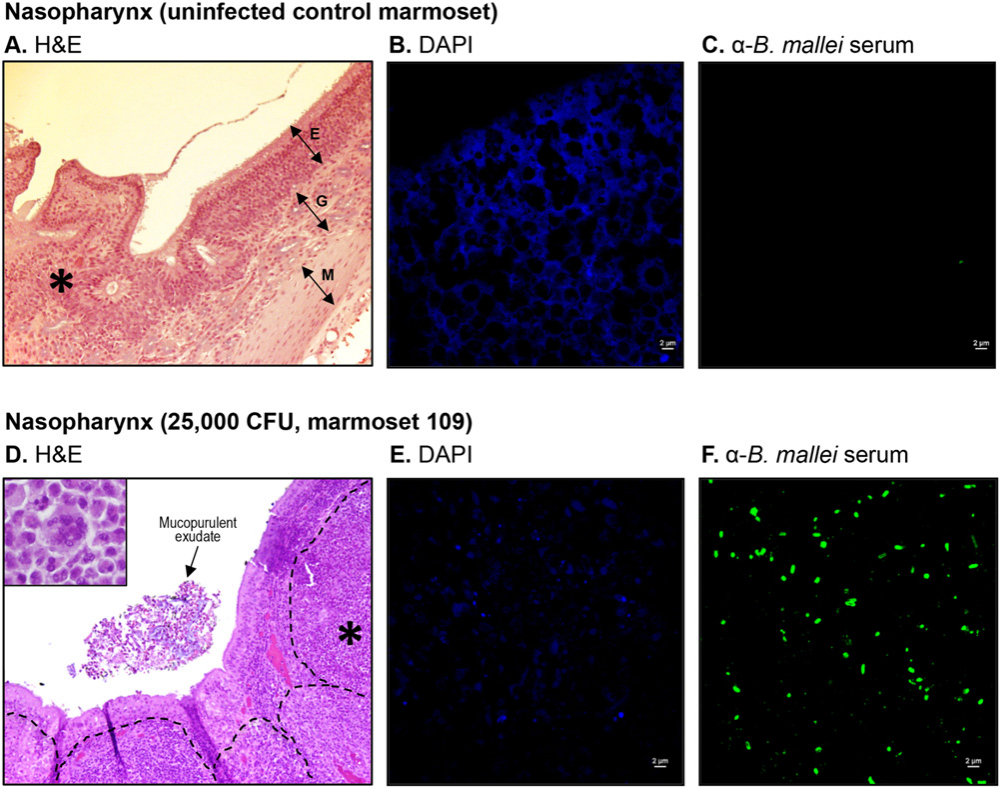
Histologic and immunofluorescence examination of nasopharyngeal tissues. Tissue sections from control and infected animals were stained with H&E (panels A and D), DAPI (panels B and E), or convalescent serum from mice that survived acute infection with *B*. *mallei* and a goat anti-mouse antibody conjugated AlexaFluor 488 (panels C and F), examined by microscopy, and photographed. Representative fields are shown. Panel A shows tissue within normal limits from an uninfected control animal at a magnification of 20x. The tissue consisted of a mucosal surface composed of ciliated pseudostratified columnar epithelial cells and goblet cells (E), supported by underlying secretory glands (G) and smooth muscle (M). The asterisk indicates the area used to acquire the images shown in panels B and C. Panel B shows staining of nucleic acid from host cells (blue) at a magnification of 100x. Panel C shows the lack of reactivity of the anti-*B*. *mallei* serum with tissue from the uninfected control animal at a magnification of 100x. Panel D shows tissues from marmoset 109, which was infected with a medium-high dose of *B*. *mallei* (25,000 CFU), at a magnification of 20x. Mucopurulent exudate was present in the nasal cavity and the tissue exhibited extensive pyogranulomatous rhinitis with marked necrosis. The dotted lines show coalescing pyogranulomas that infiltrated and expanded the connective tissues underlying/supporting the nasal mucosa. The inset shows one of many multinucleated giant cells observed in pyogranulomas at a magnification of 100x. The asterisk indicates the area used to acquire the images shown in panels E and F. Panel E shows staining of nucleic acid from host cells and bacteria (blue) at a magnification of 100x. Panel F shows reactivity of the anti-*B*. *mallei* serum with intralesional bacilli (green) at a magnification of 100x.

**Fig 3:**
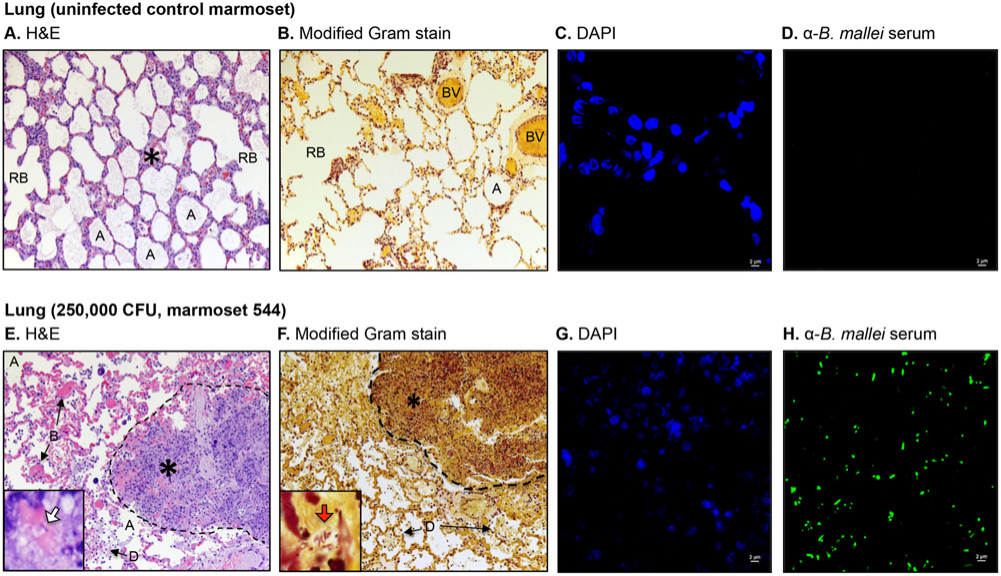
Histologic and immunofluorescence examination of lung tissues. Tissue sections from control and infected animals were stained with H&E (panels A and E), a modified Gram stain method (panels B and F), DAPI (panels C and G), or convalescent serum from mice that survived acute infection with *B*. *mallei* and a goat anti-mouse antibody conjugated AlexaFluor 488 (panels D and H), examined by microscopy, and photographed. Representative fields are shown. Panel A and B show tissue within normal limits from an uninfected control animal at a magnification of 20x. The tissue contained numerous clear alveolar spaces (A), respiratory bronchioles (RB) and blood vessels (BV). The asterisk indicates the area used to acquire the images shown in panels C and D. Panel C shows staining of nucleic acid from host cells (blue) at a magnification of 100x. Panel D shows the lack of reactivity of the anti-*B*. *mallei* serum with tissue from the uninfected control animal at a magnification of 100x. Panel E and F shows tissues from marmoset 544, which was infected with a high dose of *B*. *mallei* (250,000 CFU), at a magnification of 20x. The dotted lines delineate a severe necropurulent granuloma surrounded by clear alveolar spaces as well as alveolar spaces filled with blood (B) and/or necrotic debris (D). The inset in Panel E shows an intralesional bacillus (white block arrow) at a magnification of 100x. The inset in Panel F shows intralesional Gram negative (dark pink-red) bacilli (red block arrow) at a magnification of 100x. The asterisk indicates the area used to acquire the images shown in panels G and H. Panel G shows staining of nucleic acid from host cells and bacteria (blue) at a magnification of 100x. Panel H shows reactivity of the anti-*B*. *mallei* serum with intralesional bacilli (green) at a magnification of 100x.

**Fig 4:**
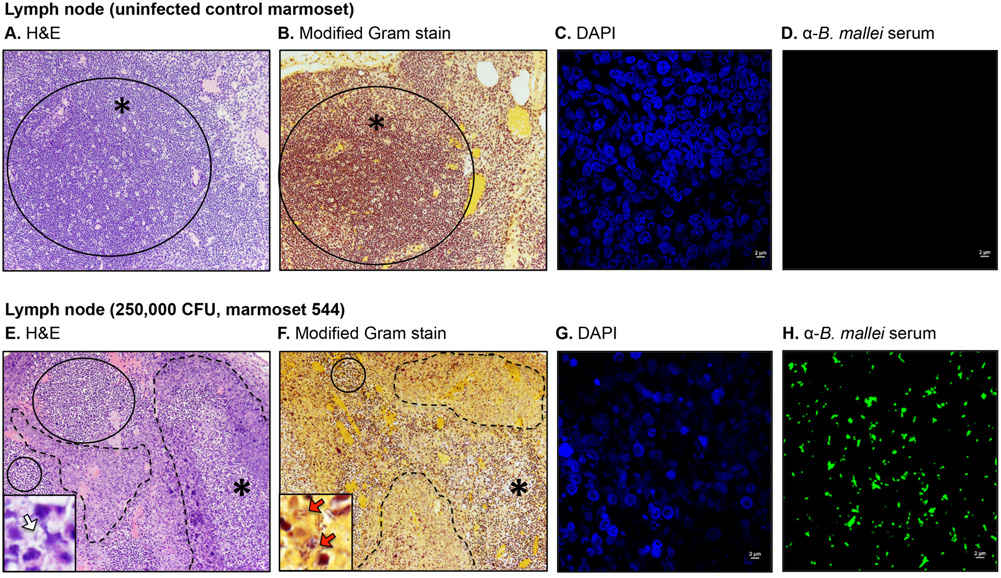
Histologic and immunofluorescence examination of lymph node tissues. Tissue sections from control and infected animals were stained with H&E (panels A and E), a modified Gram stain method (panels B and F), DAPI (panels C and G), or convalescent serum from mice that survived acute infection with *B*. *mallei* and a goat anti-mouse antibody conjugated AlexaFluor 488 (panels D and H), examined by microscopy, and photographed. Representative fields are shown. Panel A and B show tissue within normal limits from an uninfected control animal at a magnification of 20x. The solid line circle indicates a normal diffuse medullary lymphatic tissue. The asterisk indicates the area used to acquire the images shown in panels C and D. Panel C shows staining of nucleic acid from host cells (blue) at a magnification of 100x. Panel D shows the lack of reactivity of the anti-*B*. *mallei* serum with tissue from the uninfected control animal at a magnification of 100x. Panel E and F shows tissues from marmoset 544, which was infected with a high dose of *B*. *mallei* (250,000 CFU), at a magnification of 20x. The tissue contained necrotic and purulent areas (dashed lines) almost entirely effacing normal lymph node architecture (solid line circles). The inset in Panel E shows an intralesional bacillus (white block arrow) at a magnification of 100x. The inset in Panel F shows intralesional Gram negative (dark pink-red) bacilli (red block arrows) at a magnification of 100x. The asterisk indicates the area used to acquire the images shown in panels G and H. Panel G shows staining of nucleic acid from host cells and bacteria (blue) at a magnification of 100x. Panel H shows reactivity of the anti-*B*. *mallei* serum with intralesional bacilli (green) at a magnification of 100x.

**Fig 5:**
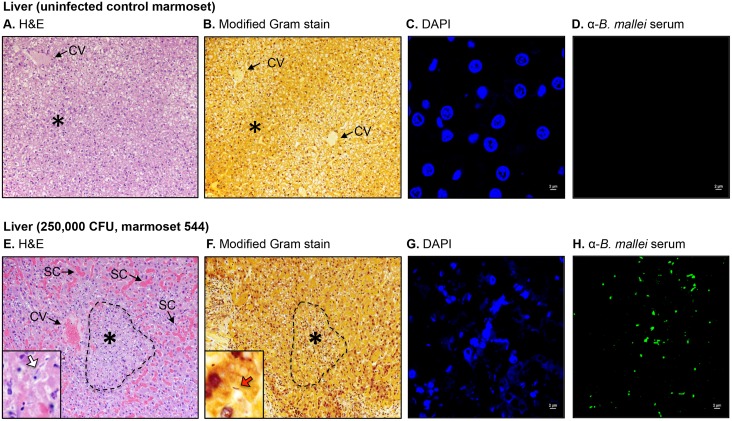
Histologic and immunofluorescence examination of liver tissues. Tissue sections from control and infected animals were stained with H&E (panels A and E), a modified Gram stain method (panels B and F), DAPI (panels C and G), or convalescent serum from mice that survived acute infection with *B*. *mallei* and a goat anti-mouse antibody conjugated AlexaFluor 488 (panels D and H), examined by microscopy, and photographed. Representative fields are shown. Panel A and B show tissue within normal limits from an uninfected control animal at a magnification of 20x. The tissues consisted of closely packed rows of hepatocytes, portal tracts and blood vessels all radiating from a central vein (CV). No prominent liver sinusoid capillaries were present. The asterisk indicates the area used to acquire the images shown in panels C and D. Panel C shows staining of nucleic acid from host cells (blue) at a magnification of 100x. Panel D shows the lack of reactivity of the anti-*B*. *mallei* serum with tissue from the uninfected control animal at a magnification of 100x. Panel E and F shows tissues from marmoset 544, which was infected with a high dose of *B*. *mallei* (250,000 CFU), at a magnification of 20x. The dashed lines indicate foci with completely disrupted liver architecture consisting of necrotic hepatocytes and leukocytes debris surrounded by an enlarged and distorted central vein (CV) as well as numerous dilated liver sinusoid capillaries (SC). The inset in Panel E shows intralesional bacilli (white block arrow) at a magnification of 100x. The inset in Panel F shows an intralesional Gram negative (dark pink-red) bacillus (red block arrow) at a magnification of 100x. The asterisk indicates the area used to acquire the images shown in panels G and H. Panel G shows staining of nucleic acid from host cells and bacteria (blue) at a magnification of 100x. Panel H shows reactivity of the anti-*B*. *mallei* serum with intralesional bacilli (green) at a magnification of 100x.

**Fig 6:**
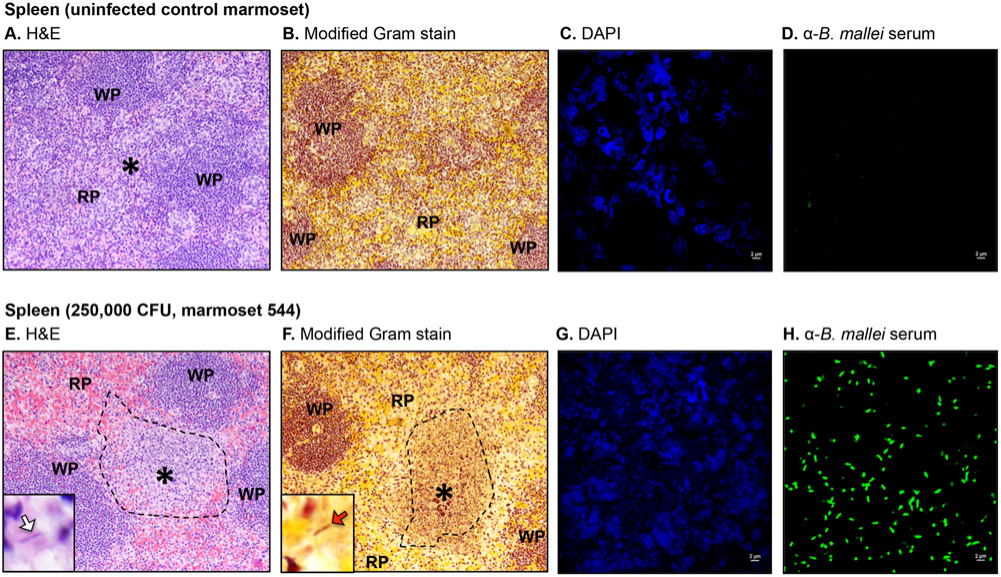
Histologic and immunofluorescence examination of spleen tissues. Tissue sections from control and infected animals were stained with H&E (panels A and E), a modified Gram stain method (panels B and F), DAPI (panels C and G), or convalescent serum from mice that survived acute infection with *B*. *mallei* and a goat anti-mouse antibody conjugated AlexaFluor 488 (panels D and H), examined by microscopy, and photographed. Representative fields are shown. Panel A and B show tissue within normal limits from an uninfected control animal at a magnification of 20x. The tissues consisted of a meshwork of white pulp (WP) containing a large number of lymphoid cells admixed with red pulp (RP). The asterisk indicates the area used to acquire the images shown in panels C and D. Panel C shows staining of nucleic acid from host cells (blue) at a magnification of 100x. Panel D shows the lack of reactivity of the anti-*B*. *mallei* serum with tissue from the uninfected control animal at a magnification of 100x. Panel E and F shows tissues from marmoset 544, which was infected with a high dose of *B*. *mallei* (250,000 CFU), at a magnification of 20x. The dashed lines indicate necropurulent areas with complete loss of normal follicular structure surrounded by normal meshworks of white and red pulp. The inset in Panel E shows an intralesional bacillus (white block arrow) at a magnification of 100x. The inset in Panel F shows an intralesional Gram negative (dark pink-red) bacillus (red block arrow) at a magnification of 100x. The asterisk indicates the area used to acquire the images shown in panels G and H. Panel G shows staining of nucleic acid from host cells and bacteria (blue) at a magnification of 100x. Panel H shows reactivity of the anti-*B*. *mallei* serum with intralesional bacilli (green) at a magnification of 100x.

To further investigate pathologic changes induced by *B*. *mallei*, tissues were collected at the indicated end points, fixed with formalin, processed into thin sections, stained, and examined by microscopy. The histology findings are summarized in Table 3 and representative images are shown in (Figs 2–6). Most pathology was observed in the lungs, liver, spleen, lymph nodes and nasopharynx, hence only these tissues are depicted. Consistent with the clinical, bacteriology and cytology findings, the tissues from animals in the low dose groups (25 and 250 CFU) did not demonstrate gross or histologic evidence of infection, similar to samples from the uninfected controls. The nasopharyngeal mucosal surface was composed of histologically normal ciliated, pseudostratified columnar epithelial cells and goblet cells, supported by underlying secretory glands and connective tissue (Fig 2A). The lungs contained numerous clear alveolar spaces and were composed of histologically unremarkable bronchi, bronchioles, alveoli, and their associated blood vessels (Fig 3A). The lymph nodes were surrounded by a thin capsule and organized into a prominent outer cortex of lymphoid follicles containing large numbers of lymphoid cells and an inner medullary layer composed of lymphocytes, rare plasma cells, and few macrophages (Fig 4A). Liver tissues consisted of closely packed rows of hepatocytes, portal tracts, and blood vessels all radiating outward from a central vein. No prominent liver sinusoid capillaries were noted (Fig 5A). The spleens were surrounded by a thin capsule and composed of an aggregated meshwork of white pulp containing large numbers of lymphoid cells, admixed with red pulp consisting of a dense meshwork of erythroid precursors, erythrocytes, and blood vessels (Fig 6A).

**Table 3:** Marmoset identifiers, groups, and gross pathology, and histopathology findings.

Identifier	Inoculating dose (CFU)	Lungs	Spleen	Liver	Nasopharynx	Lymph nodes
171	25	Normal	Normal	Normal	Normal	Normal
172	25	Normal	Normal	Normal	Normal	Normal
174	25	Normal	Normal	Normal	Normal	Normal
186	25	Normal	Normal	Normal	Normal	Normal
189	250	Normal	Normal	Normal	Normal	Normal
191	250	Normal	Normal	Normal	Normal	Normal
194	250	Normal	Normal	Normal	Normal	Normal
196	250	Normal	Normal	Normal	Normal	Normal
225	2,500	Normal	Microabscesses with scattered degenerate neutrophils	Normal	Normal	Normal
968	2,500	Normal	Normal	Normal	Normal	Normal
D65	2,500	Normal	Microabscesses with scattered degenerate neutrophils	Normal	Normal	Normal
D37	2,500	Normal	Splenomegaly with microabscesses, pyogranulomas, and degenerate neutrophils	Fibrinopurulent perihepatitis and hepatitis with degenerate neutrophils and ILB	Mucopurulent and pyogranulomatous rhinitis with degenerate neutrophils and ILB	Normal
109	25,000	Small foci of embolic pneumonia with ILB	Microabscesses with scattered degenerate neutrophils and ILB	Scattered microabscesses with necrotic hepatocytes and ILB	Pyogranulomatous rhinitis with marked necrosis, ILB, and MGC	Normal
755	25,000	Small foci of embolic pneumonia with ILB	Microabscesses with scattered degenerate neutrophils and ILB	Scattered microabscesses with necrotic hepatocytes and ILB	Pyogranulomatous rhinitis with marked necrosis and ILB	Normal
135	25,000	Normal	Normal	Normal	Normal	Normal
F48	250,000	Necropurulent pneumonia with ILB and MGC	Splenomegaly, microabscesses with scattered degenerate neutrophils	Scattered microabscesses and necrotic hepatocytes	Necropurulent and pyogranulomatous rhinitis	Normal
55B	250,000	Scattered necrotizing pyogranulomas with ILB and MGC	Splenomegaly, pyogranulomatous splenitis with ILB and MGC	Multifocal necrotizing hepatitis with ILB	Necropurulent rhinitis and osteomyelitis with ILB and fibrin thrombi	Normal
544	250,000	Severe coalescing necropurulent bronchopneumonia with ILB and MGC	Splenomegaly, multifocal and coalescing necropurulent splenitis with ILB	Multifocal necrotizing hepatitis with ILB	Necropurulent rhinitis with ILB and MGC	Necropurulent lymphadenitis with ILB

ILB = intralesional bacilli, MGC = multinucleated giant cells

Other tissues examined (kidneys, adrenal glands, stomach, duodenum, jejunum, ileum, cecum, colon) did not show gross or histologic evidence of infection

The samples obtained from marmosets D37, 225 and D65 in the medium dose group (2,500 CFU) contained lesions indicative of infection. Marmoset D37 had severe bilateral mucopurulent and pyogranulomatous rhinitis, focally-extensive fibrinopurulent perihepatitis and hepatitis, and marked splenomegaly with microabscesses and pyogranulomas. All lesions contained numerous degenerate neutrophils, and the nasopharyngeal and liver specimens showed intralesional bacilli. These findings are consistent with the obvious symptoms of illness displayed by the animal on day 8 post-challenge (Table 1), colonization of its spleen and liver with *B*. *mallei* (Table 1), and the pathologic changes observed in splenic and hepatic impression smears (Table 2). Interestingly, the spleens of marmosets D65 and 225, which were culture negative and exhibited non-specific signs of illness (Table 1), contained microabscesses with scattered degenerate neutrophils. The nasopharyngeal and liver samples from these 2 animals appeared normal. The fourth marmoset in this group, 968, did not show gross or histological evidence of infection, correlating with the animal’s unremarkable clinical presentation (Table 1), culture negative status (Table 1), and the normal appearance of liver, lungs and spleen tissue imprints (Table 2). The lungs and lymph nodes samples from all 4 marmosets in the medium dose group were within normal limits.

Examination of tissues from marmosets 109 and 755 in the medium-high dose group (25,000 CFU) revealed lesions in the nasopharynx, lungs, spleen, and liver with intralesional bacilli (Table 3). These findings correlate with the obvious symptoms of infection displayed by these animals on day 4 post-challenge (Table 1), colonization of their tissues with *B*. *mallei* (Table 1), and the pathologic changes observed in impression smears (Table 2). Both animals showed small foci of embolic pneumonia and pyogranulomatous rhinitis with marked necrosis (Table 3). The nasopharyngeal lesion in marmoset 109 was particularly extensive with inflammatory cells and bacilli infiltrating the surrounding subcutis, skeletal muscle, bone marrow, lymphatics, and blood vessels (Fig 2D); immunofluorescence staining of the tissue confirmed the intralesional bacilli as *B*. *mallei* (Fig 2F). Multinucleated giant cells, which are hallmarks of infection with the organism observed both *in vitro* and *in vivo* [2, 40–42], were also present (inset in Fig 2D). The liver and spleen of both marmosets (109 and 755) contained multiple microabscesses with necrotic parenchymal cells and degenerate neutrophils, but lymph nodes were within normal limits (Table 3). Consistent with the clinical and bacteriology findings (Table 1), the tissues from the third marmoset in the medium-high dose group, 135, did not demonstrate pathology.

Tissues from all three marmosets in the high dose group (250,000 CFU) were examined and showed extensive pathology in their nasal cavity, lungs, liver and spleen with intralesional bacilli and multinucleated giant cells (Table 3). The most prominent lesions in the nasopharynx were necrosis of the mucosa accompanied by pyogranulomatous inflammation and mucopurulent exudate within the nasal cavity. In marmoset 55B, small amounts of mucopurulent exudate with moderate numbers of degenerate neutrophils were also present in the larynx. Pulmonary lesions varied from small, scattered parenchymal necropyogranulomas in marmosets 55B and F48, to severe and massive necropurulent bronchopneumonia in marmoset 544. Panels E-H of Fig 3 depict a representative necropurulent granulomatous lesion and identify intralesional bacilli as *B*. *mallei*. Examination of liver samples from marmoset 544 revealed numerous, variably-sized, and scattered foci of necrosis composed of leukocyte nuclear debris, necrotic hepatocytes, and intralesional *B*. *mallei* bacilli (Fig 5E–5H). The liver tissues from marmosets F48 and 544 showed scattered microabscesses with minimally damaged parenchyma, and necrosis of the myeloid lineage in hepatic foci of extramedullary hematopoiesis, respectively (Table 3). Grossly, the spleens of all three marmosets in the high dose group were markedly enlarged and contained 1–2 mm white scattered lesions (data not shown). Histologically, splenic lesions varied from scattered microabscesses (F48, Table 3), large multifocal pyogranulomas with multinucleated giant cells (55B, Table 3), to multifocal and coalescing necropurulent splenitis (544, Table 3 and Fig 6E–6H). Only one marmoset in this group, 544, showed pathology in lymph node tissues. Fig 3 depicts sections of tracheobronchial lymph nodes from this animal containing necrotic and purulent lesions that almost entirely efface normal lymph node architecture. Taken together, these histological findings are consistent with the severe clinical signs of glanders displayed by marmosets F48, 55B and 544 on days 3 and 4 post-challenge (Table 1). The pathology in tissues of marmoset F48 also correlates with cytology findings (Table 2) and colonization of the lungs, spleen and liver with *B*. *mallei* (Table 1).

## Discussion

In this study, we demonstrate that the common marmoset is susceptible to intranasal infection with *B*. *mallei*. We show that this non-human primate develops an illness similar to that described in other established models for the organism, and the disease onset and progression resemble that reported for humans with glanders. We also document the median lethal dose that causes acute disease by the intranasal route of inoculation and detail pathologic changes induced by *B*. *mallei* during infection.

We discovered that 91% of animals infected with doses ranging from 25 to 2.5 X 10^3^ bacteria exhibited mild and non-specific signs of illness and were culture negative for *B*. *mallei*. Only one marmoset inoculated with 2.5 X 10^3^ organisms (D37, Table 1) developed moderate signs of disease, reached humane end-points 8 days post-infection, and was culture positive. Challenge experiments also revealed that 83% of animals inoculated with higher doses of 2.5 X 10^4^ and 2.5 X 10^5^ bacteria developed acute lethal infection within 3–4 days. Only one marmoset inoculated with 2.5 X 10^4^ organisms (135, Table 1) was culture negative and presented with mild and non-specific symptoms. Based on these results, we calculated a median lethal dose for *B*. *mallei* strain ATCC 23344 of 9,953 CFU. This value is higher than that reported for other models of *B*. *mallei* respiratory infection, but this finding is not that surprising. It is known that the choice of bacterial strain, inoculation route, and animal background can significantly affect the course of disease. For example, infection of BALB/c mice with *B*. *mallei* ATCC 23344 via the intranasal or intratracheal route produced LD_50_ values of ~ 800 organisms [31, 37], while the median lethal dose of the strain upon intraperitoneal inoculation is 7 X 10^5^ CFU [27]. In contrast, the LD_50_ for the agent via the intraperitoneal route is less than 10 bacteria in hamsters [28, 43]. Our study is the first to provide a median lethal dose for *B*. *mallei* in marmosets, hence no direct comparison can be made with data from other groups. A marmoset model of acute aerosol infection has been established for the closely-related bacterium *Burkholderia pseudomallei*, the causative agent of melioidosis [44]. However, the authors of that study were not able to establish an LD_50_ value as animals inoculated with as few as 2 CFU reached humane end-points 78-hr upon challenge. In those experiments, marmosets were inoculated using an aerosol chamber.

We observed that marmosets infected with higher doses of *B*. *mallei* develop an acute onset of clinical signs (within 24-hr) and rapidly progressive lethal disease (3–8 days), the severity of which correlates with inoculum size. Symptoms in animals infected with medium, medium-high, and high doses consisted of subdued behavior (decreased vocalizations and limp tails), lethargy, inappetence, decreased water intake, decreased grooming behavior with piloerection (rough coats), ocular and nasal discharges, conjunctivitis, tachypnea (increased respiratory rate), and dyspnea (difficulty breathing with abdominal lifts). Natural cases of glanders in humans and other animals exhibit many of these signs, namely malaise, excessive lacrimation (tearing), nasal discharge, conjunctivitis, tachypnea, and dyspnea [1, 6, 7, 12–16, 18, 45, 46]. These hallmarks also have been reported in murine and equine experimental models of glanders [26, 27, 31].

Consistent with their clinical presentation, the marmosets infected with higher doses of *B*. *mallei* were colonized with the organism (Table 1). Though discrepancies between bacteriology and histology findings were noted, these were likely caused by collecting separate portions of the same tissues for analysis. For example, the lungs of marmoset 109 were culture negative (Table 1), but contained lesions with bacilli on histology (Table 3). These lesions were not grossly visible and appeared focal in distribution by histology. Hence, portions of tissues lacking lesions were presumably and inadvertently sampled to determine bacterial burden. Another possible explanation for discrepancies between bacteriology and histology findings is that the number of organisms in tissues was below the limit of detection of our culture method. The negative culture obtained for the lungs of another marmoset that developed overt signs of illness, D37, is not surprising since no pathologic lesions were found by cytology (Table 2) or histopathology (Table 3). This finding is counterintuitive given that lung colonization was expected after intranasal inoculation. However, glanders is notorious for its varied clinical presentation. In natural cases of infection, it is not uncommon to see localized disease, disseminated disease, or even local disease that rapidly progresses to dissemination [1, 6, 7, 12–16, 18, 45, 46]. Marmoset D37 had obvious nasopharyngeal lesions (Table 3), thus we suspect that bacteria disseminated directly from these lesions to the liver and spleen via the circulatory system. This trafficking mechanism is especially likely since *B*. *mallei* is known for having a predilection for reticuloendothelial organs [27–29, 31, 38], as these organs are highly vascularized and utilize large numbers of resident macrophages to survey and filter the blood during circulation.

To our knowledge, this is the first study in which impression smears from affected organs were examined cytologically for evidence of infection with *B*. *mallei*. Our laboratory developed a mouse model of intratracheal inoculation for the organism, and we reported bacteriology and cytopathologic analyses of tissues [31]. However, the samples analyzed in that particular study were from bronchoalveolar lavages (BAL). The primary pathologic change noted in the murine BAL samples was the presence of suppurative (neutrophilic) inflammation characterized by a predominance of degenerate neutrophils with both intracellular and extracellular bacilli. These changes were identified in impression smears of the spleen and liver of marmosets infected with medium and high doses of *B*. *mallei* (D37 and F48, respectively). Additional findings in these samples consisted of highly vacuolated and degranulated neutrophils, band neutrophils, lymphoid hyperplasia, and increased extramedullary hematopoiesis (Table 2). Lymphoid hyperplasia is indicative of antigenic stimulation, which can be attributed to bacterial infection and subsequent inflammatory response occurring in tissues. Increased and/or excessive extramedullary hematopoiesis is often seen in animals with disseminated infections, particularly those with agents eliciting excessive inflammatory responses. It should be noted that 2 marmosets, 109 and 755, showed histologic lesions (Table 3) and/or were colonized with *B*. *mallei* (Table 1) in the liver and spleen, but lacked pathologic changes on cytology (Table 2). Additionally, all lung samples obtained and evaluated by cytology appeared normal despite the presence of histologic lesions and culture positive results in some marmosets. These discrepancies are likely attributed to sampling error as some of these lesions were not grossly visible and appeared mild and focal in their distribution by histology. Histopathology on tissues from marmosets infected with *B*. *mallei* consisted of necrotizing purulent to pyogranulomatous rhinitis, pneumonia, splenitis, hepatitis, and lymphadenitis. These findings are consistent with those described in other established experimental models for glanders including mice, hamsters, and horses. Moreover, these pathologic changes are also the classic lesions documented for cases of *B*. *mallei* infection in humans, horses, and camels [1, 3–7, 11, 13–18, 26, 40]. We observed Gram-negative bacilli intimately associated with nearly all necrotizing lesions in marmoset tissues, and these bacilli were specifically identified as *B*. *mallei* by immunofluorescence staining.

While preparing this manuscript, Nelson and colleagues published a report in which the common marmoset was used to develop a subcutaneous model of infection for *B*. *mallei* [47]. Marmosets were inoculated in the inner thigh with 10^2^ CFU of strain ATCC 23344 and reached humane endpoints 7–10 days post-challenge. In contrast, the marmosets in our study did not demonstrate clinical evidence of glanders and lethality until challenged with 10^3^ organisms. We determined a median lethal dose of 9,953 CFU, with animals reaching humane endpoints within 3–8 days. This difference in bacterial dosage and survival most likely reflects the different routes of inoculation used. Subcutaneous injection circumvents physical barriers employed by the immune system to combat infection and allows immediate access to host cells as well as blood and lymphatic vessels. Conversely, the intranasal route of inoculation exposes *B*. *mallei* to the respiratory mucosal barrier and mucociliary escalator, which the organism has to overcome in order to establish itself and cause disease. Similar to our findings, marmosets inoculated subcutaneously exhibited clinical signs (lethargy, rough coat, conjunctivitis) and histopathology (necrotizing pneumonia, small pyogranulomas in the liver and spleen) documented in other experimental models of glanders as well as in natural cases in equids and humans. Hence our findings complement those of Nelson and colleagues and underscore the usefulness and relevance of the marmoset model to study glanders and develop countermeasures.
